# Cholecystectomy: Advances and Issues

**DOI:** 10.3390/jcm11123534

**Published:** 2022-06-20

**Authors:** Raimundas Lunevicius

**Affiliations:** Department of General Surgery, Liverpool University Hospitals NHS Foundation Trust, Lower Lane, Liverpool L9 7AL, UK; raimundas.lunevicius@liverpoolft.nhs.uk

The introduction and rationalization of the terms ‘*Critical View of Safety*’ (CVS) in 1995–2000 [1] and ‘*Culture of Safety in Cholecystectomy*’ (COSIC) in 2014–2020 [2] constitute the most remarkable advancement in cholecystectomy theory and practice since the first laparoscopic removal of the gallbladder in the Böblingen county hospital on 12 September 1985 [3]. The latter term encircles a variety of well-defined objective (inflammatory changes, detection of cystic structures, bail-out strategies, etc.) and less-well-defined subjective (e.g., timely decision-making to conduct a few momentary pauses during difficult gallbladder operation and reconsider or discuss the concreteness related to situation and anatomy) elements within the whole scheme of surgical safety.

In 2020, the COSIC initiative materialised into the evidence-based safe cholecystectomy guideline consensus recommendations for surgical practice [2]. It also included recommendations for future studies as only 2 out of 13 recommendations were classified as having a strong scientific base—first, for use of intraoperative biliary imaging (in particular, intraoperative cholangiography) in the event of uncertain biliary anatomy or suspicion of bile duct injury (BDI) and, second, referral of patients with confirmed or suspected BDI to the experienced surgeon or multispecialty hepatobiliary team. Of note, 154 studies were selected to inform this guideline after assessing 765 full-text records for eligibility. Insufficient quality of papers and records with duplicate patient cohorts or without data amenable for synthesis were dominant reasons for exclusion of 611 articles. That should be considered when planning new research or audit studies to add further value for and enhance COSIC.

The impact on general surgeons of one of the key requirements of this conception—to use the CVS method of the cystic artery and cystic duct identification during cholecystectomy—was tremendous. The method has been endorsed by numerous surgical societies and groups of general surgeons as its application increases the patient’s safety during laparoscopic cholecystectomy. However, there is another side of the coin of CVS: the application of alternative surgical strategies and methodical actions when a safe exposure and detection of both cystic structures becomes problematic.

Difficult surgical situations in gallbladder surgery for a benign disease are uncommon compared to the whole cholecystectomy patient population. When surgeons encounter them and realise that a total cholecystectomy is not safely achievable, the less risky ‘bail-out’ method should be considered. Subtotal cholecystectomy is one of the few bail-out surgical procedures. A systematic review of all published papers on subtotal cholecystectomy on risks associated with this operation reported results from 85 clinical studies conducted in 29 countries from six continents between January 1985 and June 2020 [4]. Forty-five of these studies (52.9%) were carried out in Japan, India, the UK, and the US. Seventy-two (84.7%) were published between 2000 and 2020. On one hand, these data strongly suggest that subtotal cholecystectomy (as a recognised bail-out action when the structures of the cystic pedicle cannot be identified safely) is a part of armamentarium of gallbladder surgeons from individual institutions. On the other hand, information extracted from synthesis of 85 studies is insufficient even to speculate on nationwide patterns of trends in alternative gallbladder surgery.

Epidemiological studies on the provision of gallbladder surgical procedures in individual countries are of paramount importance as they assess national trends to understand the challenges of the present. For example, between 2000 and 2019, a sevenfold increase in subtotal cholecystectomy rates was detected in England [5]. Consequently, a fivefold decrease in the ratio of total to subtotal cholecystectomy was reported in the country, dropping from 180:1 in 2000 to 38:1 in 2019. A study conducted in the United States reported a similar trend, namely an increase in the rates of open subtotal and laparoscopic subtotal cholecystectomy for acute cholecystitis [6]. These findings show that the provision of definitive surgery for a symptomatic or complicated benign biliary disease, especially in England, has declined.

Therefore, broad unanswered questions remain. What are the reasons for the apparent shift toward subtotal cholecystectomy in England? Is it related to technical challenges to achieve CVS and, if so, why? What is the amplitude of the bias estimating the means with associated uncertainty intervals for rates of subtotal cholecystectomies? Does the increase in subtotal cholecystectomy reciprocally correlate with the incidence of BDI over time? It should, according to the culture of safe cholecystectomy scheme; however, is it the case? Is it possible to model and forecast the future of gallbladder surgery for benign diseases? What are the trends and patterns within these trends in gallbladder surgery in other parts of the world? What are the barriers (if any) within national health services or local authorities to implement the recommendations from numerous guidelines, such as Tokyo, the Association of Upper Gastrointestinal Surgery of Great Britain and Ireland, the National Institute for Health and Care Excellence, the World Society of Emergency Surgery, and the Society of American Gastrointestinal and Endoscopic Surgeons, on safe management of symptomatic and complicated gallbladder stone diseases? Additionally, what are the barriers to documenting every suspected or proven injury to the bile ducts and registering it accordingly within national administrative data bases and (or) surgical society specific registries?

The answers to these questions require a standardised estimation based on a linkage to big data from International Statistical Classification of Diseases and Related Health Problems and Office of Population Censuses and Surveys’ Classification of Interventions and Procedures. At present, it is not feasible to determine whether the increase in subtotal cholecystectomy reciprocally correlates with the incidence of BDI in England over time as there are no codes for iatrogenic injuries to the extrahepatic bile ducts. The data of the national snapshot audits can only provide valuable results and insights into the BDI problem. CholeS Study Group publications are a good example [7]. Thirteen patients in the cholecystectomy intra-operative difficulty grades 4 and 5 had injuries to the extrahepatic bile ducts for an injury rate of 1.7%, which is 56 and 10 times higher than grade-1 and grade-3 difficulty-specific BDI rates, respectively, and almost seven times higher than the overall BDI rate of 0.25%. To emphasize, a high intra-operative difficulty grade is one of the reasons for conversion from total cholecystectomy to subtotal cholecystectomy to mitigate BDI risk during gallbladder surgery.

The surrogate codes are not the best solution for estimation of BDI rates, although analyses based on such codes provide important information on magnitude and trends in major BDI requiring an anastomosis during reconstructive surgery. For example, a paper published in 2013 reported a rate of major BDI of 0.4% in England between 2000 and 2009, which was stable over time [8]. However, it must be noted that most cholecystectomy associated injuries to the extrahepatic bile ducts do not require bilioenteric anastomosis. Thus, controversy remains over the estimation of the incidence of BDI of all grades.

Another direction for future research aiming to provide evidence on how to reduce the risk or severity of BDI is multi-institutional studies with a narrower focus to address specific research or audit questions or nuances regarding the preoperative and intraoperative risk stratification, efficacy and effectiveness of new surgical techniques and technologies, cholecystectomy for patients from specific or higher-risk subpopulations, basic and advanced educational programs to promote the individual learning, and systematic framework-based analyses of errors.

There is no doubt that intraoperative findings will surpass the information available to surgeons before surgery. However, it is crucial to predict the cholecystectomy course in advance, especially when a second opinion regarding laparoscopic or open subtotal cholecystectomy, cholecystolithotomy, or abandoned cholecystectomy may be required. Identifying clinical-radiological predictors of difficult cholecystectomy and producing cholecystectomy-specific preoperative risk assessment tools is important for clinicians.

An intraoperative fluorescent cholangiography method using indocyanine green (tricarbocyanine dye) and a near-infrared light source in laparoscopic cholecystectomy is an example of a new imaging method despite a long history of indocyanine green utilisation in liver surgery. It is important to emphasize that indocyanine green has only one contraindication for the standard dose of less than 2 mg/kg [9]. It is hypersensitive to iodine or other substances that contain iodine. The optimal indocyanine green dosage, administration timing, and mode before laparoscopic cholecystectomy to reduce the fluorescence ratio is far from being standardised [10]. The patient and inflammatory pathology-related factors in which indocyanine green provides a benefit through increased safety in laparoscopic cholecystectomy should be better understood. To the best of our knowledge, intraoperative indocyanine is superior to white light alone in identifying the extrahepatic biliary anatomy, thus decreasing the risk of BDI [10]. Further study is needed on clinical outcomes of laparoscopic cholecystectomy associated with intraoperative fluorescent cholangiography, although this intraoperative method for ductal visualisation corresponds well with the conception of COSIC.

The number and size of ports for laparoscopic cholecystectomy is another common theme among surgeons/scientists. Indeed, less invasiveness in laparoscopic cholecystectomy correlates with decreased blood loss, reduced postoperative analgesia and length of stay in hospital, faster recovery, earlier return to work, and improved cosmesis. However, the analysis of the indications for mini-laparoscopic cholecystectomies reveals that minimalistic techniques in laparoscopic cholecystectomy are feasible only for the least difficult cholecystectomies. Examples of these indications include gallbladder polyps, dyskinesia, microlithiasis, and cases of pancreatitis where no cause has been identified. To note, the use of 10-, 5-, 3-, and 2-mm trocars is currently recommended for mini-laparoscopic cholecystectomies. Their minimal number is discussed in the literature as it has the potential to augment the inherent benefits of minimal access surgery. Further trials will help ascertain potential advantages of minimalistic or super-minimalistic approaches for laparoscopic cholecystectomy. Transabdominal single incision laparoscopic surgery, robot-assisted surgery, natural orifice transluminal endoscopic surgery, gasless cholecystectomy, and hybrid procedures are part of this. The critical point in assessing the studies remains the same: BDI incidence as the primary outcome. Cosmesis should be regarded as the last proxy outcome.

Previously, non-operative management of symptomatic cholecystolithiasis in pregnancy has been recommended. However, early elective laparoscopic cholecystectomy is the treatment of choice for pregnant individuals with this disease, regardless of trimester [11]. The items of informed consent of a pregnant individual should be documented thoroughly. Therefore, the gallbladder surgeon must be informed about the result of a urine pregnancy test on the day of planned surgery.

Informed consent for cholecystectomy of pregnant individuals is one of the examples of traditional surgical ethics from the surgeon–patient perspective [12]. Introducing new technics and technologies for laparoscopic cholecystectomy requires balancing the potential medical and non-medical harms and expected benefits. An example of the non-medical harm after cholecystectomy is patient-reported outcomes. To date, we have no firm knowledge on the quality of life of patients who underwent subtotal cholecystectomy. Additionally, during the informed consent process, it should be explained why the surgeon plans to use a novel technique as this is required under the same ethical principle (respect for patient autonomy) [13].

Ethical dilemmas based on the surgeon–colleague perspective have not been investigated comprehensively. For example, the correctness of operation notes is of paramount importance as it is a source of information for medical professionals (to facilitate best patient care) as well as administrative (to code the surgical procedure precisely), scientific (to collate surgery-specific data prospectively or retrospectively), and judicial (in the event of inquiries into the allegations) applications. The provision of incorrect documentation on difficult or complicated cholecystectomy can be interpreted as inappropriate conduct (misconduct) towards other members of the surgical team (see *Good Surgical Practice* at https://www.rcseng.ac.uk/standards-and-research/gsp/, accessed on 15 June 2022). A possible scenario of such practice is that of a gallbladder surgeon asked for a second opinion and technical help in the event of perforation of the right anterior sectional bile duct (proven via intraoperative cholangiography) who documents the injury to the right hepatic bile duct (which was uninjured) on the operation note.

Last, the gallbladder and bile duct disease-specific personal healthcare access and quality index (HAQ index) is a new outcome measure from the system-public perspective. It is based on amenable mortality, defined as deaths from an acute or chronic biliary disease that should not occur in the presence of timely and effective care. It is worth analysing results for 195 countries and territories from the Global Burden of Disease study 2016 [14]. However, it must be appreciated that, in 2016, the UK ranked 63rd worldwide on the HAQ index specific to biliary disease management with a score of 81 out of 100. Such suboptimal performance in a state with a free publicly funded healthcare system strongly suggests that further urgent research is warranted to generate causal models and valuable explanations for this phenomenon.

In brief, advances in clinical and epidemiological knowledge and surgical and engineering techniques should revolve around one axis: a universal culture of patient safety. The three-decade history of laparoscopic cholecystectomy demonstrates that individual initiative-based collaborative work is the guarantor of achieving the best results globally. However, the end of this work is behind the horizon.
